# Fluor Albus

**Published:** 1876-03

**Authors:** J. G. Westmoreland

**Affiliations:** Atlanta, Ga.


### FLUOR ALBUS.

By J. G. WESTMORELAND, M.D., Atlanta, Ga.

Leucorrhœa and fluor albus literally mean a white flow or flux. These terms have been applied to a mucus discharge from the vagina, and is commonly known as *whites*. Blennorrhœa, with a prefix denoting the part from which mucus flows, is equally applicable, and even more appropriate, if the white discharge be mucus. Thus vaginal blennorrhœa would be understood as mucus discharge from the vagina.

Now is this mucus discharge from the vagina entitled to the dignity of a disease, which may be relieved by a particular remedy applied to the vagina, or by any particular class of what are called general remedies, introduced into the circulation? We unhesitatingly answer no. In the first place, it must be remembered that the discharge may originate in the uterus, the urethra or in the vagina itself, and may depend upon relaxation, irritation or inflammation of the mucous surface from which the flow proceeds. Not only so, but if inflammation gives rise to it, there must be some practical importance in ascertaining whether it be specific or non-specific, chronic or acute.

Leucorrhœa, fluor albus, or whites, or more practically speaking, a white discharge from the vagina, may originate in the mucous membrane of that canal, and depend upon merely an inactive, relaxed, or passive state of tissue, requiring only the application of a decoction of oak bark, or solution of tannin, twice daily, for its relief. If, however, the discharge proceeds from the interior of the uterus, no benefit can result from this or other treatment directed to the vagina.

The writer had indulged the hope that such terms as leucorrhœa, diarrhœa, blennorrhœa, prolapsus, and dropsy, though once thought sufficiently definite, had been entirely discarded by modern writers, except in giving the result or symptom of disease. There is as much propriety in calling all diseases of the respiratory organs broncorrhœa, because expectoration results from them, as in naming and treating all diseases of the female procreative organs on the supposition that all the trouble exists in the vagina producing too much mucus, or the uterus being too low in the pelvis. With such nomenclature and such treatment I had abundant reason to be disgusted at least twenty years ago. If young men now entering the profession have to be taught by rote the particular remedies for diarrhœa, dropsy, fluor albus, and prolapsus uteri, the progress in medicine for the last quarter of a century is not worth a fig! Years of laborious investigation and experiment have gone for naught to the rising generation of doctors! If no useful and practical advancement has been made in the treatment of female diseases during the last twenty-five years, I am very clear in the opinion that the best treatment for the suffering female is the advice to take no treatment at all. Blood, digestive, and nervous tonics, with pessaries and vaginal washes, have made many dollars for the doctors, but very little health for the women.

Female diseases, particularly those of the womb, are certainly treated more successfully since practitioners have become more critical in determining the particular location and character of disease, and by rational means impressing diseased parts where ever they are found. The profession of medicine has advanced beyond the point where prostration, hysteria, palpitation, and all the nervous pains and aches resulting from local organic lesions of the stomach, liver, uterus, etc., are treated for impoverished blood, indigestion, and nervous debility. It is now known that these symptoms often exist without any very prominent manifestation of disease discovered in the organ at fault; and it is only after critical examination of various organs that the true cause is discovered. It is also known that vigor to the nervous and other systems of the body is restored by *curing* the local disease, where ever it may be.

The profession of medicine is not one grand mutual admiration society, to praise and please each other, but each member should aid in pathological and practical advancement, without jealousy or unkind feeling toward colaborers.

